# Bronchoscopy to assess patients with hemoptysis: which is the optimal timing?

**DOI:** 10.1186/s12890-019-0795-9

**Published:** 2019-02-11

**Authors:** Michele Mondoni, Paolo Carlucci, Giuseppe Cipolla, Alessandro Fois, Stefano Gasparini, Silvia Marani, Stefano Centanni, Giovanni Sotgiu

**Affiliations:** 1 Department of Health Sciences, Respiratory Unit, ASST Santi Paolo e Carlo, Università degli Studi di Milano, San Paolo Hospital, Via Di Rudinì n. 8, 20142 Milan, Italy; 2 ASST Lodi, UOC Pneumologia, Lodi, Italy; 3 Lung Disease Unit, Department of Medical, Surgical, Experimental Sciences, University of Sassari, Sassari, Italy; 4 Department of Biomedical Sciences and Public Health, Università Politecnica delle Marche, Ancona, Italy; 5 Pulmonology Unit, AOU “Ospedali Riuniti”, Ancona, Italy; 6 ASST Mantova, Dipartimento Cardio-Toraco-Vascolare, Unità Operativa di Pneumologia e UTIR, Mantova, Italy; 7 Clinical Epidemiology and Medical Statistics Unit, Department of Medical, Surgical, Experimental Sciences, University of Sassari, Sassari, Italy

**Keywords:** Bronchoscopy, Hemoptysis, Lung cancer, Bronchiectasis, Pneumonia

## Abstract

**Background:**

Bronchoscopy plays a key role to diagnose the etiology, to localize the site, and to identify the sources of the bleeding in patients with hemoptysis, but the ideal timing of an endoscopic examination is still unclear.

**Methods:**

We performed a secondary analysis of an observational and multicenter study, aimed at evaluating the epidemiology of hemoptysis in Italy and the diagnostic yield of the most frequently prescribed examinations. The aim of the study was to evaluate whether an early bronchoscopy (i.e., performed during active bleeding/≤48 h after hemoptysis stopped) helps localize bleeding (i.e., site, lobe, lung) and increase diagnostic yield in comparison with a delayed examination.

**Results:**

Four hundred eighty-six consecutive adult patients (69.2% males; median [IQR] age: 67 [53–76] years) with hemoptysis requiring an etiological diagnosis and undergoing bronchoscopy were recruited.

Bleeding focus could be located more frequently in case of moderate-severe bleedings than in cases of mild hemoptysis (site: 70/154, 45.4%, VS. 73/330, 22.1%; *p*-value < 0.0001; lobe: 95/155, 61.3%, VS. 95/331, 28.7%; p-value < 0.0001; lung: 101/155, 65.1%, VS. 111/331, 33.5%; *p*-value < 0.0001). Early bronchoscopy showed a higher detection rate of bleeding source in comparison with delayed examination (site: 76/214, 35.5%, VS. 67/272, 24.6%; *p*-value = 0.01; lobe: 98/214, 45.8%, VS. 92/272, 33.8%; p-value = 0.007; lung: 110/214, 51.4%, VS. 102/272, 37.5%; p-value = 0.002). Early bronchoscopy did not provide any advantages in terms of increased diagnostic yield, in the total cohort (113/214, 52.8%, VS. 123/272, 45.2%; p-value = 0.10) and in the severity subtypes (mild: 56/128, 43.8%, VS. 88/203, 43.4%; p-value = 0.94; moderate-severe: 57/86, 66.2%, VS. 35/69, 50.7%; p-value = 0.051).

**Conclusions:**

Early bronchoscopy helps detect bleeding sources, particularly in cases of moderate-severe hemoptysis, without increasing diagnostic accuracy.

**Trial registration:**

ClinicalTrials.gov (identifier: NCT02045394).

## Background

Hemoptysis is a challenging symptom associated with potentially life-threatening medical conditions [1–3]. A recent European, observational study showed that malignancies were the most frequent etiology [3]. On this basis, diagnostic work-up should be as comprehensive as possible [3, 4].

Detection of bleeding sites is key for a successful clinical management, particularly in patients with life-threatening bleeding [5, 6].

Computed tomography (CT) and bronchoscopy are accurate techniques for diagnosis and localization of bleeding sources [1–3, 7, 8]. However, in comparison with CT, which is more accurate for the diagnosis of vascular and parenchymal disorders, bronchoscopy could better assess upper airways and endobronchial abnormalities (e.g.*,* endobronchial malignancies), as well as it can provide histopathological and microbiological samples from central and peripheral lung lesions [3, 9–16]. Furthermore, it may be useful in patients requiring endobronchial interventions, and in case of bilateral lung abnormalities where radiographic localization of a bleeding source might be challenging [5, 6].

No guidelines exist on the optimal timing of diagnostic bronchoscopy in patients with hemoptysis [5]. Only a few studies assessed whether the timing of endoscopy may affect the identification and the diagnosis of bleeding sources [17, 18]. Their findings were controversial and, consequently, the ideal timing continues to remain a matter of debate [5, 17, 18].

The aim of our study was to evaluate whether an early bronchoscopy (i.e., performed during active bleeding/≤48 h after hemoptysis stopped) helps detect bleeding sources and increase diagnostic yield (i.e. ability to provide histopathological and/or microbiological specimens useful for an etiological diagnosis) in comparison with a delayed (i.e., performed after 48 h hemoptysis subsided) examination [17].

We also investigated whether symptom’s severity might influence the ability of the endoscopic examination to localize bleeding focus.

## Methods

This was a secondary analysis of an observational and multicenter study, aimed at evaluating epidemiology of hemoptysis in Italy and diagnostic yield of the most frequently prescribed diagnostic examinations [3] . The study protocol was approved by ethical committees of five Italian participating hospitals (Milan, Mantua, Lodi, Sassari, Ancona) and registered at ClinicalTrials.gov (identifier: NCT02045394). Written informed consent was signed by recruited patients [3].

From July 2013 to September 2015, consecutively recruited adult (i.e., ≥18 years old) patients with hemoptysis requiring an etiological diagnosis underwent bronchoscopy. Exclusion criteria were the following: 1) etiology of hemoptysis already found; 2) refusal to sign the informed consent [3].

Severity of hemoptysis was assessed by the attending physician considering the daily amount of expectorated blood: mild (from some drops of blood to 20 ml (ml)/24 h –h-), moderate (20–500 ml/24 h), severe (> 500 ml/24 h) [1–3, 6].

Bronchoscopic examination was considered positive only if it proved an endobronchial bleeding lesion and/or provided histopathological and/or microbiological specimens, helpful for a definitive etiological diagnosis [3, 17]. Furthermore, we evaluated the ability of bronchoscopy to detect the bleeding source (i.e., anatomic site, lobe, and lung). The site of the bleeding was defined as the exact visible hemorrhage source (e.g., a visible bleeding endobronchial malignancy). Direct visualization of active bleeding/oozing was considered suggestive of a bleeding source [17, 19]. During the analysis patients undergoing bronchoscopy were divided into two groups on the basis of the timing of bronchoscopy in relation with hemoptysis interruption. The 48-h cut-off was chosen according to the only available study which evaluated the same topic (i.e. diagnostic yield and ability of bronchoscopy to localize the bleeding source in relation to the time of the intervention) [17].

No pre-defined endoscopic protocols were planned owing to the lack of evidence-based recommendations [5, 7]. Decision to perform bronchoscopy, timing of bronchoscopy, type of bronchoscope (flexible, rigid), and/or positioning of an endotracheal tube were evaluated by the attending physician case by case after careful assessment of clinical features, previous individual experiences, and availability of instruments.

Life-threatening hemoptysis was defined as any hemoptysis where blood loss was > 100 mL in a time-frame of 24 h, causing abnormal gas exchange/airway obstruction and/or hemodynamic instability. Notably, daily blood loss > 100 ml (i.e. moderate or severe hemoptysis in our study) is the smallest amount of blood loss reported in scientific literature to potentially cause a life-threatening medical condition [20].

In our study patients with moderate and severe hemoptysis were combined in a single group. Indeed, as previously stated, life-threatening hemoptysis might have occurred only in case of moderate or severe bleeding (daily blood loss > 100 ml).

Qualitative and quantitative variables were collected and summarized with absolute and relative (percentages) frequencies and medians (interquartile ranges, IQR) in case of non-parametric distribution, respectively. Qualitative variables were compared using chi-squared or Fisher exact test when appropriate. A two-tailed, *p*-value less than 0.05 was considered statistically significant. The statistical software used for the computations was Stata13.0 (StataCorp, College Station, TX, USA).

## Results

A total of 486 adult patients (69.2% males; median [IQR] age: 67 [53–76] years) with hemoptysis were enrolled.

Hemoptysis volume was self-reported in 416/606 (68.6%) cases, whereas it was estimated by healthcare professionals in 190/606 (31.4%).

In moderate-severe hemoptysis 87/486 (17.9%) bronchoscopy was performed within 48 h hemoptysis stopped, while 69/486 (14.2%) after 48 h. In mild hemoptysis 128/486 (26.3%) endoscopic examinations were performed within 48 h, while 202/486 (41.6%) were performed after 48 h.

471/486 (96.9%) bronchoscopies were performed with a flexible bronchoscope, 2/486 (0.4%) with a flexible bronchoscope during endotracheal intubation (moderate bleedings), and 13/486 (2.7%) with a rigid scope. Flexible bronchoscope was used in 7/12 (58.3%) severe, 134/144 (93.0%) moderate, and 330/330 (100%) mild bleedings. Rigid bronchoscopy was performed in 5/12 (41.6%) severe and 8/144 (5.5%) moderate hemoptysis. In 20/486 (4.1%) patients, hemoptysis was considered potentially life-threatening. In case of life-threatening hemoptysis all bronchoscopies were performed as soon as possible (all during active hemoptysis - within 24 h hemoptysis subsided).

Bronchoscopy was performed with a diagnostic and therapeutic (e.g., administration of topical vasoconstriction, Fogarty balloon, argon plasma coagulation, and laser) aim in 99/486 (20.4%) patients.

Demographic, epidemiologic, clinical, and endoscopic characteristics of the cohort are shown in Table 1.

**Table 1:** Demographic, clinical and bronchoscopic characteristics of the enrolled cohort

Total cohort, n (%)		486 (100)
Age (years), median (IQR)		67 (53–76)
Males, n (%)		336/486 (69.2%)
Hemoptysis severity, n (%)	Mild	330/486 (67.9%)
	Moderate	144/486 (29.6%)
	Severe	12/486 (2.4%)
Life-threatening hemoptysis, n (%)	20/486 (4.1%)	
Flexible bronchoscopies, n (%)	471/486 (96.9%)	
Flexible bronchoscopies during endotracheal intubation, n (%)	2/486 (0.4%)	
Rigid bronchoscopies, n (%)	13/486 (2.7%)	
Therapeutic bronchoscopies, n (%)	99/486 (20.4%)	

Bleeding focus localization occurred more frequently in case of moderate or severe bleedings in comparison with mild hemoptysis cases (site: 70/154, 45.4%, VS. 73/330, 22.1%; *p*-value < 0.0001; lobe: 95/155, 61.3%, VS. 95/331, 28.7%; p-value < 0.0001; lung: 101/155, 65.1%, VS. 111/331, 33.5%; p-value < 0.0001).

**Fig. 1:**
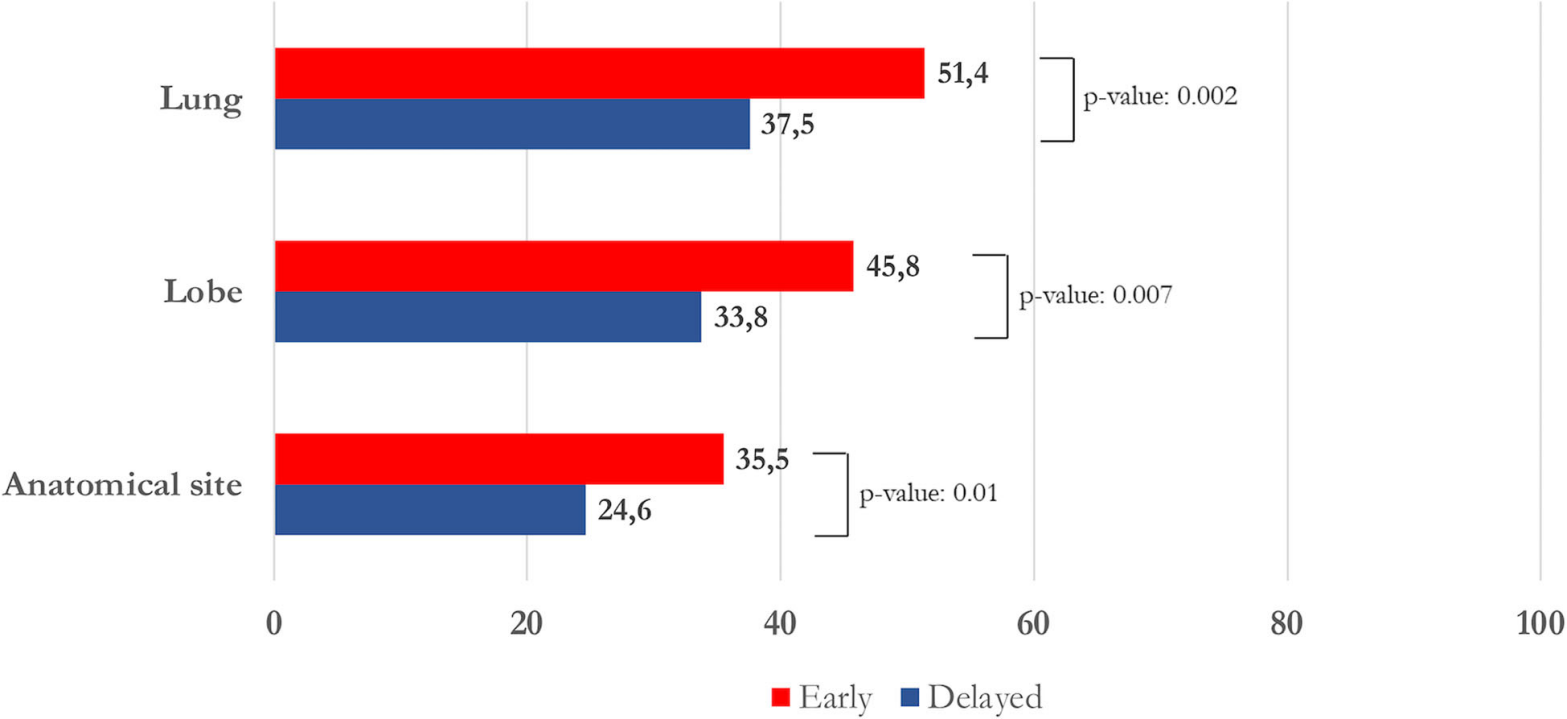
Detection rate of the bleeding source (anatomical site, lobe, and lung) in association with the bronchoscopic timing

Early bronchoscopy showed a higher bleeding source detection rate in comparison with delayed examination (site: 76/214, 35.5%, VS. 67/272, 24.6%; *p*-value = 0.01; lobe: 98/214, 45.8%, VS. 92/272, 33.8%; p-value = 0.007; lung: 110/214, 51.4%, VS. 102/272, 37.5%; p-value = 0.002) (Fig. 1). However, no differences were found when bronchoscopy was performed within 24 VS. within 48 h (site: 39/116, 33.6%, VS. 37/98, 37.8%; *p*-value = 0.52; lobe: 56/116, 48.3%, VS. 42/98, 42.9%; p-value = 0.43; lung: 63/116, 54.3%, VS. 47/98, 48.0%; p-value = 0.35).

In the subgroup of patients with mild hemoptysis, an early bronchoscopy did not show a higher ability to detect the source of the bleeding in comparison with a delayed one (site: 33/128, 25.8%, VS. 40/202, 19.8%; p-value = 0.20; lobe: 43/128, 33.6% VS. 52/203, 25.6%; p-value = 0.12; lung: 51/128, 39.8% VS. 60/203, 29.6%; p-value = 0.05).

In patients who underwent both CT and bronchoscopy, CT showed a significantly higher ability to detect the exact site of bleeding than bronchoscopy, both in early (130/252 (51.6%) VS. 73/190 (38.4%), p-value = 0.006) and delayed examinations (111/293 (37.9%) VS. 65/261 (24.9%); p-value = 0.001).

Bronchoscopy showed an overall diagnostic yield of 48.7% (237/487).

The most prevalent definitive clinical diagnosis after complete work-up where bronchoscopy showed positive findings (i.e. provided histopathological and/or microbiological specimens useful for an etiological diagnosis) were pulmonary malignancy (97/112, 86.6%;), pneumonia (31/78, 39.7%), bronchiectasis (25/65, 38.5%), and acute bronchitis (20/65, 30.8%).

In 6/65 (9.2%) patients with a final diagnosis of bronchiectasis, the bleeding source was localized only by bronchoscopy (inconclusive bilateral findings at CT scan). In 59/65 (90.7%) bronchoscopy was performed to collect microbiological/cytological samples, showing positive findings in 25/59 (42.4%) patients.

**Fig. 2:**
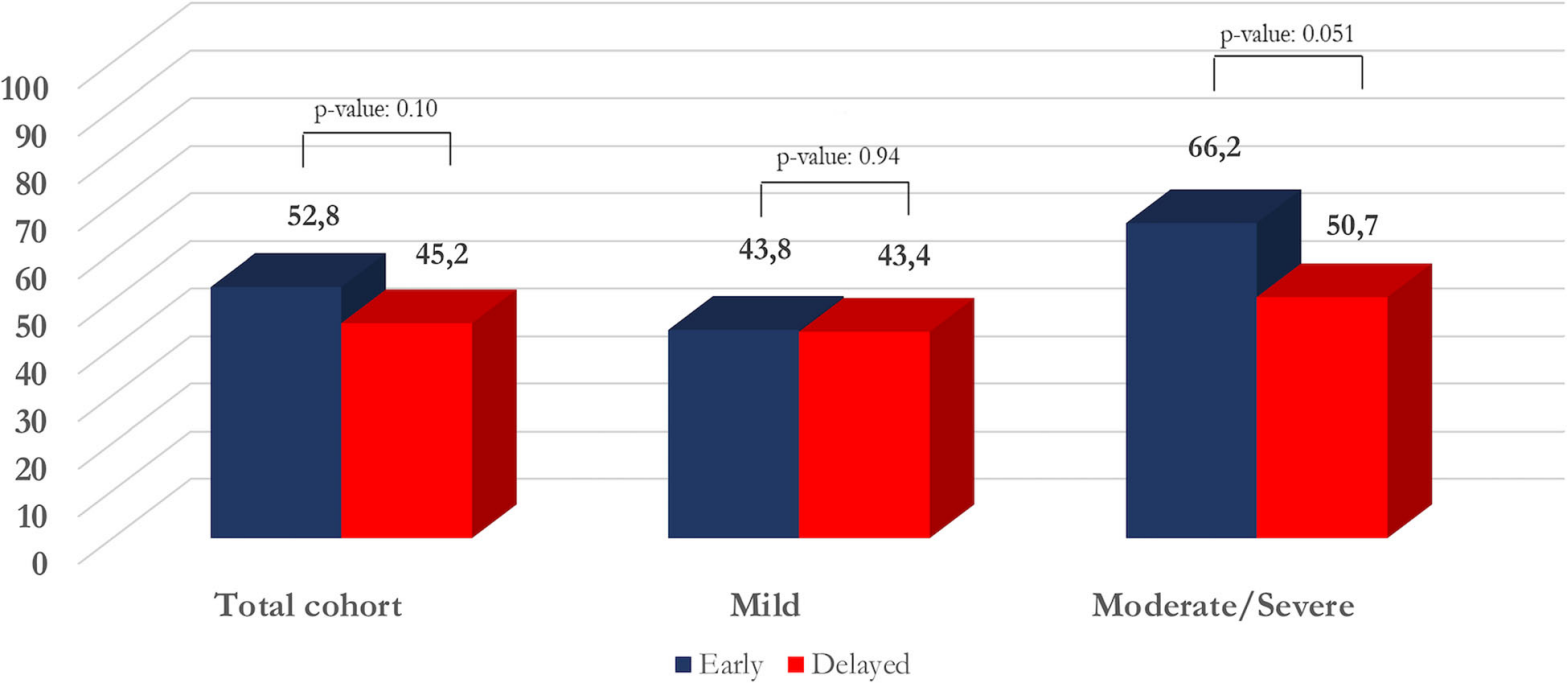
Assessment of hemoptysis etiology and timing of endoscopic examination

Early and delayed bronchoscopy did not show relevant differences in terms of diagnostic yield, for the total cohort (113/214, 52.8%, VS. 123/272, 45.2%; *p*-value: 0.10) and between severity groups (mild: 56/128, 43.8%, VS. 88/203, 43.4%; p-value: 0.94; moderate-severe: 57/86, 66.2%, VS. 35/69, 50.7; p-value: 0.051) (Fig. 2).

With regard to the main definitive diagnosis, early bronchoscopy was not associated with a significantly higher yield (malignancies: 42/45, 93.3%, VS. 55/67, 82.1%, p-value: 0.09; pneumonia: 13/28, 46.4%, VS. 18/50, 36%, p-value: 0.37; bronchiectasis: 15/32, 46.9%, VS. 10/33, 30.3%, p-value: 0.17; acute bronchitis: 13/37, 35.1%, VS. 7/28, 25%, p-value: 0.38).

Bronchoscopy was performed in 9/11 (81.8%) patients with hemoptysis who had a final diagnosis of upper airways bleeding. Notably, it showed pathological findings and detected the exact bleeding focus in all of them (9/9, 100.0%).

## Discussion

This secondary analysis, which involves a large cohort of patients, was conducted to evaluate the diagnostic accuracy of bronchoscopy for hemoptysis etiology and for bleeding source detection in relation with endoscopic timing. As previously demonstrated by Hirshberg et al., our results showed that the bleeding source detection rate can be higher in case of moderate-severe hemoptysis [1]. This finding is key in case of life-threatening events, when an accurate localization of the bleeding focus is needed to administer endoscopic therapies and/or to guide angiographic embolization [5–7].

We also demonstrated that bronchoscopy can better detect bleeding sources (i.e., anatomical site, lobe, and lung) when it is performed within 48 h from the last hemoptysis episode. This is significantly relevant for patients with moderate and severe bleeding. Indeed, when the mild group is considered alone, we failed to detect any differences between early and delayed examinations.

Notably, an investigation performed within 24 h hemoptysis subsided failed to improve the detection rate in the total cohort in comparison with a bronchoscopy performed within 48 h.

Interestingly, a bronchoscopy performed within 48 h after hemoptysis interruption does not significantly increase the diagnostic yield, regardless of symptom severity and final diagnosis.

Few studies have evaluated the most appropriate timing for diagnostic bronchoscopy in patients with hemoptysis [17, 18].

Gong et al. retrospectively analyzed a cohort of 129 patients with hemoptysis and showed that an early examination (i.e., bleeding subsided less than 48 h prior to the bronchoscopy) provided a higher bleeding detection rate; however, diagnostic yield and patient management did not improve [17]. Hsiao et al. showed in a group of non-cancer patients with moderate-severe hemoptysis that bronchoscopy performed within 24 h from the first bleeding episode was associated with a significantly higher detection rate of the bleeding source [18].

Our findings, based on a larger prospective and multicenter study, confirmed the results of Gong et al. [17]. In comparison with their findings, we detected a higher proportion of focal bleeding sites, probably owing to the higher prevalence of neoplasms (mostly endobronchial) in our cohort. Moreover, as observed by Gong et al., we could not detect active bleeding in the majority of the cases and we could not assess a better endoscopic ability in localizing bleeding sources associated with a very early evaluation [17].

Several study limitations should be acknowledged.

The observational nature of the study can increase the risk of a selection bias; however, the lack of evidence-based guidelines cannot support the design of an interventional, randomized control study for ethical reasons.

No guidelines exist on symptom severity [3, 7, 20]. As previously reported, we grouped patients on the basis of their daily blood volume loss, without considering hemodynamic consequences and gas exchange impairment/airway obstruction [1–3, 6]. Life-threatening hemoptysis may depend on wide volume ranges of expectorated blood, as well as it may depend on other clinical variables (i.e., rate of bleeding, airways blood clearance, extent and severity of any underling lung and/or cardiac disease) [5, 20].

In case of life-threatening hemoptysis, airways patency should be immediately preserved; in this context, rigid bronchoscopy or tracheal intubation are better options in comparison with flexible bronchoscopy [5, 21]. Following ventilation recovery, flexible instrument may be used for diagnosis, localization of the bleeding source, and therapy [5].

We did not evaluate if different endoscopic timing might affect clinical management. This secondary analysis was aimed at the ideal timing of diagnostic endoscopy, without considering its role on long-term outcomes.

We demonstrated that bleeding source detection rate is higher in case of moderate-severe hemoptysis. It should be underscored that in critical cases of moderate-severe hemoptysis (i.e. life-threatening hemoptysis) bronchoscopy was always performed earlier if compared with cases of non-critical bleeding. This issue might have affected the findings of the study.

Notably, the optimal timing of bronchoscopy may have less clinical relevance nowadays than in the past, given the availability of highly sensitive imaging techniques. Indeed, as suggested by our findings and by recent studies, CT imaging and bronchoscopy may have similar accuracy in the identification of the location of the bleeding, while CT may show higher sensitivity in the etiologic diagnosis and is key for bronchial artery embolization, which remains the cornerstone for the management of severe hemoptysis cases [7, 22–24].

## Conclusions

Bronchoscopy can be crucial for patients with hemoptysis. An examination performed within 48 hemoptysis stopped can help detect the bleeding source (particularly in moderate-severe hemoptysis cases), without increasing its diagnostic accuracy and regardless of the definitive diagnosis. An endoscopic examination performed within 24 h the symptom subsided does not further improve the detection rate of bleeding sources than a bronchoscopy performed within 48 h.

On the basis of these findings, in case of moderate-severe hemoptysis, if bronchoscopy is deemed crucial or CT imaging is not available or not useful to detect the bleeding source, early endoscopy could be recommended. In this context, an accurate localization of the bleeding focus is needed to rapidly administer the most adequate therapy. In case of mild bleeding, a delayed examination may be considered; the ability of bronchoscopy to diagnose underlying diseases and to localize the bleeding source are not influenced by the timing of the investigation.

## Abbreviations

CT: Computed tomography
h: Hours
IQR: Interquartile ranges
ml: Milliliters

## References

[1] Hirshberg B, Biran I, Glazer M, Kramer MR (1997). Hemoptysis: etiology, evaluation, and outcome in a tertiary referral hospital. Chest.

[2] Tsoumakidou M, Chrysofakis G, Tsiligianni I, Maltezakis G, Siafakas NM, Tzanakis N (2006). A prospective analysis of 184 hemoptysis cases: diagnostic impact of chest X-ray, computed tomography, bronchoscopy. Respiration.

[3] Mondoni M, Carlucci P, Job S, Parazzini EM, Cipolla G, Pagani M (2018). Observational, multicentre study on the epidemiology of haemoptysis. Eur Respir J.

[4] Mondoni M, Sferrazza Papa GF, Sotgiu G, Carlucci P, Pellegrino GM, Centanni S (2016). Haemoptysis: a frequent diagnostic challenge. Eur Respir J.

[5] Sakr L, Dutau H (2010). Massive hemoptysis: an update on the role of bronchoscopy in diagnosis and management. Respiration.

[6] Grosu HB, Casal RF, Morice RC, Nogueras-González GM, Eapen GA, Ost D (2013). Bronchoscopic findings and bleeding control predict survival in patients with solid malignancies presenting with mild hemoptysis. Ann Am Thorac Soc.

[7] Gagnon S, Quigley N, Dutau H, Delage A, Fortin M (2017). Approach to hemoptysis in the modern era. Can Respir J.

[8] McGuinness G, Beacher JR, Harkin TJ, Garay SM, Rom WN, Naidich DP (1994). Hemoptysis: prospective high-resolution CT/bronchoscopic correlation. Chest.

[9] Thirumaran M, Sundar R, Sutcliffe IM, Currie DC (2009). Is investigation of patients with haemoptysis and normal chest radiograph justified?. Thorax.

[10] Mondoni M, Carlucci P, Cipolla G, Fois A, Gasparini S, Marani S (2018). Bronchoscopy in patients with hemoptysis and negative imaging tests. Chest.

[11] Cardenas-Garcia J, Feller-Kopman D (2018). POINT: should all initial episodes of hemoptysis be evaluated by bronchoscopy? Yes. Chest.

[12] Cardenas-Garcia J, Feller-Kopman D (2018). Rebuttal from Drs Cardenas-Garcia and Feller-Kopman. Chest.

[13] Mondoni M, Sotgiu G, Bonifazi M, Dore S, Parazzini EM, Carlucci P (2016). Transbronchial needle aspiration in peripheral pulmonary lesions: a systematic review and meta-analysis. Eur Respir J.

[14] Rivera MP, Mehta AC, Wahidi MM (2013). Establishing the diagnosis of lung Cancer. Chest.

[15] Mondoni M, Carlucci P, Di Marco F, Rossi S, Santus P, D’Adda A (2013). Rapid on-site evaluation improves needle aspiration sensitivity in the diagnosis of central lung cancers: a randomized trial. Respiration.

[16] Mondoni M, Radovanovic D, Valenti V, Patella V, Santus P (2015). Bronchoscopy in sarcoidosis: union is strength. Minerva Med.

[17] Gong H, Salvatierra C (1981). Clinical efficacy of early and delayed fiberoptic bronchoscopy in patients with hemoptysis. Am Rev Respir Dis.

[18] Hsiao EI, Kirsch CM, Kagawa FT, Wehner JH, Jensen WA, Baxter RB (2001). Utility of Fiberoptic bronchoscopy before bronchial artery embolization for massive hemoptysis. Am J Roentgenol.

[19] Seon HJ, Kim Y-H, Kwon Y-S (2016). Localization of bleeding sites in patients with hemoptysis based on their chest computed tomography findings: a retrospective cohort study. BMC Pulm Med.

[20] Ibrahim WH (2008). Massive haemoptysis: the definition should be revised. Eur Respir J.

[21] Haponik EF, Chin R (1990). Hemoptysis: clinicians’ perspectives. Chest.

[22] Chalumeau-Lemoine L, Khalil A, Prigent H, Carette MF, Fartoukh M, Parrot A (2013). Impact of multidetector CT-angiography on the emergency management of severe hemoptysis. Eur J Radiol.

[23] Nielsen K, Gottlieb M, Colella S, Saghir Z, Larsen KR, Clementsen PF (2016). Bronchoscopy as a supplement to computed tomography in patients with haemoptysis may be unnecessary. Eur Clin Respir J.

[24] Bønløkke S, Guldbrandt LM, Rasmussen TR (2015). Bronchoscopy in patients with haemoptysis and normal computed tomography of the chest is unlikely to result in significant findings. Dan Med J.

